# Detection of *Streptococcus pneumoniae*, *Neisseria meningitidis* and *Haemophilus influenzae* in Culture Negative Cerebrospinal Fluid Samples from Meningitis Patients Using a Multiplex Polymerase Chain Reaction in Nepal

**DOI:** 10.3390/idr13010019

**Published:** 2021-03-01

**Authors:** Supriya Sharma, Jyoti Acharya, Dominique A. Caugant, Megha Raj Banjara, Prakash Ghimire, Anjana Singh

**Affiliations:** 1 Central Department of Microbiology, Tribhuvan University, Kirtipur, Kathmandu 44600, Nepal; banjaramr@gmail.com (M.R.B.); prakashghimire@gmail.com (P.G.); anjanas67@gmail.com (A.S.); 2 National Public Health Laboratory, Teku, Kathmandu 44600, Nepal; jyotigan30@gmail.com; 3 WHO Collaborating Centre for Reference and Research on Meningococci, Norwegian Institute of Public Health, 0213 Oslo, Norway; dominiqueandreeyvette.caugant@fhi.no

**Keywords:** bacterial meningitis, culture, polymerase chain reaction, Nepal

## Abstract

The rapid identification of bacteria causing meningitis is crucial as delays in the treatment increase mortality rate. Though considered as the gold standard for the laboratory diagnosis of bacterial meningitis, culture might give false negative results in a case of patients under antibiotics prior to lumbar puncture. This study aimed to detect *Streptococcus pneumoniae*, *Neisseria meningitidis* and *Haemophilus influenzae* by a multiplex polymerase chain reaction (PCR) in culture-negative cerebrospinal fluid samples collected from clinically suspected meningitis cases attending different hospitals in Kathmandu, Nepal from January 2017 to December 2019. *S. pneumoniae*, *N. meningitidis* and *H. influenzae* were detected in 8.59% (33/384) of the specimens by PCR and 7.55% (29/384) of the specimens by culture. Correlation between culture and PCR of the same sample was good (Spearman’s rho correlation coefficient = 0.932). However, the difference in positivity between culture and PCR was statistically not significant (*p* value > 0.05). In four specimens, culture could not detect any of the targeted bacteria whereas PCR could detect presence of *H. influenzae*. PCR increases the diagnostic yield for bacterial meningitis. PCR may be considered as an adjunctive test for establishing the cause of infection in culture negative clinically suspected meningitis cases.

## 1. Introduction

Bacterial meningitis is a very serious condition with death potentially occurring within few hours of the onset of symptoms, if untreated [1].The laboratory diagnosis of bacterial meningitis in low-income countries such as Nepal is based on Gram’s staining, culture and microscopic and biochemical analysis of cerebrospinal fluid (CSF) samples. Though considered to be the gold standard, CSF culture takes time and might delay diagnosis. Moreover, bacterial growth rate is low particularly in the CSF of patients who have already received antibiotics before lumbar puncture [2]. On the other hand, microscopic and biochemical analysis of CSF gives only indicative but not definitive identification of bacterial pathogens. Furthermore, sensitivity of Gram’s staining of CSF varies considerably for different types of bacteria [3,4]. Hence, in most of the circumstances, empirical antibiotic treatment is usually initiated immediately on the basis of clinical findings. However, for effective treatment, the bacterial pathogens causing meningitis should be rapidly identified.

Delays in the diagnosis can be avoided through the use of a molecular method such as polymerase chain reaction (PCR) which can detect small amounts of bacterial DNA independently from the growth of bacteria causing meningitis [5,6,7]. PCR is highly sensitive and specific and can detect the bacteria in CSF in patients who had used antibiotics prior to lumbar puncture [8]. *Streptococcus pneumoniae*, *Neisseria meningitidis* and *Haemophilus influenzae* are the most commonly reported bacteria causing meningitis [9]. These three bacterial pathogens are fastidious and may not survive long transit times or fluctuations in temperature during transportation of CSF specimens to a microbiology laboratory [10]. Multiplex PCR offers the advantage of using a minimal CSF sample compared to uniplex PCR for the simultaneous detection of three pathogens in a single reaction [11]. The sample volume required for each run is considered important in case of precious samples such as CSF which is difficult to obtain in large amounts. Therefore, this study aimed to detect *S. pneumoniae*, *N. meningitidis* and *H. influenzae* by a multiplex PCR in culture negative CSF samples collected from clinically suspected meningitis cases in Kathmandu, Nepal.

## 2. Materials and Methods

### 2.1. Study Sites

Nepal is a landlocked country in Asia that which between China in the north and India in the south, east and west. Kathmandu, the capital city of Nepal is densely populated with diverse ethnic groups and culture. The study sites were the major hospitals within Kathmandu valley which included Bhaktapur Hospital, Bir hospital, Kanti Children’s Hospital (KCH), Sukraraj Tropical and Infectious Diseases Hospital (STIDH) and Tribhuvan University Teaching Hospital (TUTH). The study sites receive referrals of patients from all over Nepal.

### 2.2. Study Design

This study was conducted on CSF samples collected from clinically suspected meningitis cases attending different hospitals from January 2017 to December 2019. The clinically suspected meningitis cases were selected on the basis of WHO case definition as any person with sudden onset of fever (>38.5 °C rectal or 38.0 °C axillary) and one of the following signs: neck stiffness, altered consciousness or other meningeal sign [9]. 

### 2.3. Specimen Collection

The CSF sample was collected by the attending physician/medical officer by lumbar puncture (LP) from each clinically suspected meningitis case at the respective study site. A sterile wide bore needle was inserted between the lumbar vertebrae L4 and L5 and the CSF sample was allowed to drip into the sterile container [12].Altogether 387 CSF samples were processed for culture immediately after collection and stored at −80 °C. As we aimed to compare the detection of *N. meningitidis*, *S. pneumoniae* and *H. influenzae* in CSF by two methods, three samples positive for *E. coli* were excluded in this study. Hence, 384 samples negative by culture (*n* = 355) and positive for *N. meningitidis*, *S. pneumoniae* and *H. influenzae* (*n* = 29) were processed by PCR.

### 2.4. Culture

A loopful of specimen was inoculated immediately within 30 min into blood agar and chocolate agar (Hi Media Laboratories, Pvt. Limited, India) plates and incubated in candle jar (5–10% CO_2_) at 37 °C for 24 h. As these fastidious bacteria grow well in a humid atmosphere, a dampened paper towel was kept at the bottom of the candle jar. The moisture source was changed regularly to prevent contamination with molds [9]. Identification of bacterial isolates was done at National Public Health Laboratory (NPHL), Teku, Kathmandu, Nepal by standard microbiological techniques including observation of colony characteristics, Gram’s staining, catalase, oxidase and other biochemical tests [13]. 

### 2.5. DNA Extraction

Extraction of bacterial genomic DNA from CSF sample was done by using QIAamp DNA mini kit 250 (Cat No. 51306, Lot No. 148022423) (Qiagen, Germany) following manufacturer’s instructions [14]. The quantitation of DNA in each sample was done by using QubitTM dsDNA BR Assay kit (Ref No. Q32850, Lot No. 1910794) (Invitrogen by Thermo Fisher Scientific, USA) following manufacturer’s instruction [15]. 

### 2.6. PCR

The extracted DNA (10pg) was processed at Central Department of Microbiology, Tribhuvan University, Kirtipur, Kathmandu, Nepal for multiplex PCR to detect *ctrA* gene (*N. meningitidis)*, *plyA* gene *(S. pneumoniae)* and *bex* gene *(H. influenzae*) as described elsewhere [16]. Briefly, the forward and reverse primers (Macrogen Inc., Seoul, Korea) used were GCTGCGGTAGGTGGTTCAA andTTGTCGCGGATTTGCAACTA respectively for *ctrA* gene, TGCAGAGCGTCCTTTGGTCTAT and CTCTTACTCGTGGTTTCCAACTTGA respectively for *plyA* gene and TATCACACAAATAGCGGTTGGand GGCCAAGAGATACTCATAGAACGTT, respectively, for *bex* gene. PCR mixtures contained 21.0 μL hotstart mastermix (Qiagen, Germany), 0.5 μL of 0.5 μM each primer (Macrogen Inc., Korea) and 1.0 μL DNA template. Amplification was performed in a 25 μL reaction volume using a thermocycler (Applied Biosystems), with initial activation for 15 min at 95 °C followed by 35 cycles of denaturation for 25 s at 95 °C, primer annealing for 40 s at 57 °C and DNA extension for 1 min at 72 °C, and then final extension for 5 min at 72 °C. 

### 2.7. Agarose Gel Electrophoresis

**Figure 1:**
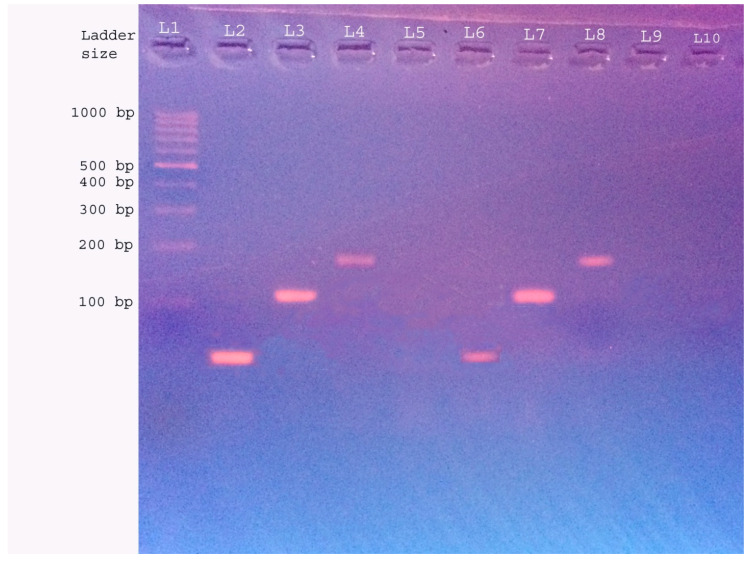
Bands visualized under UV fluorescence following agarose gel electrophoresis of PCR products (Lane1—marker 100 bp DNA, L2—positive control (PC) of *bex*(80 bp). L3—PC of *ctrA*(110 bp), L4—PC of *plyA*(181 bp), L5—No Template Control, L6—*bex*positive. L7—*ctrA* positive, L8—*plyA* positive, L9 and L10—Samples negative for *ctrA*, *bex* and *plyA.*

Amplicons of 110 bp, 80 bp and 181 bp, respectively, of *ctrA*, *bex* and *plyA* were visualized under UV fluorescence following electrophoresis in 3.5% agarosegel stained with ethidium bromide (Merck). A 100 bp DNA ladder (ThermoFisher Scientific, Waltham, MA, USA), positive controls of DNA from *S. pneumoniae* ATCC 49619, *H. influenzae* ATCC 49247 and *N. meningitidis* Z1503 cultures as well as no template controls were included during each run (Figure 1).

### 2.8. Data Analysis

The obtained data were entered into Microsoft office Excel 2007 and analyzed using IBM Statistical Package for Social Sciences (SPSS) version 21 software. Correlation between culture and PCR of the same sample was calculated using Spearman’s rho correlation coefficient. The difference in positivity between culture and PCR was statistically calculated using chi-square test. A *p*-value of <0.05 was considered statistically significant. Available laboratory investigations of culture negative but PCR positive CSF samples were noted from hospital records and analyzed. A cut-off value of CSF cell count (>5 WBCs/mm^3^), decreased glucose concentration (<45 mg/dL) and increased protein concentration (>45 mg/dL) were considered significant for the diagnosis of bacterial meningitis [9].

## 3. Results

### 3.1. Detectionof Bacteriain CSF

Among 384 cases, 56.77% were male. The highest number of samples was from neonates (Table 1).

*S. pneumoniae*, *N. meningitidis* and *H. influenzae* were detected in 8.59% (33/384) by PCR and 7.55% (29/384) by culture (Table 2). 

Correlation between culture and PCR of the same sample was good (Spearman’s rho correlation coefficient = 0.932). However, the difference in positivity between culture and PCR was statistically not significant (*p* value > 0.05). Four culture negative specimens were positive for *Haemophilus influenzae* by PCR (Table 3).

About 20% (77/384) of cases had received antibiotics prior to lumbar puncture (LP). Among them, meningococcal (*n* = 1) and pneumococcal (*n* = 1) cases were detected by both PCR and culture. But, three cases with prior antibiotics receipt were detected only by PCR (Table 3).

### 3.2. Laboratory Analysis of Culture Negative But PCR Positive Meningitis Cases

CSF samples from culture negative but PCR positive *H. influenzae* meningitis cases were also positive by acridine orange stain and had low glucose concentration, high protein concentration and cell count > 5 WBCs/mm^3^ (Table 4).

## 4. Discussion

The rapid identification of bacteria causing meningitis is crucial as delays in the treatment increase mortality rate [17]. Though traditionally considered as gold standard, the sensitivity of CSF culture is diminished in case of patients under antibiotic treatment prior to lumbar puncture. This hinders definitive treatment of cases [18]. Such a limitation could be overcome by the molecular approach. In our previous multicentric hospital-based study on bacterial meningitis which was based on conventional culture techniques, there was low culture yield of CSF [12]. To improve the diagnosis of bacterial meningitis in culture negative CSF samples, we employed multiplex PCR for the detection of *S. pneumoniae*, *N. meningitidis* and *H. influenzae* in the CSF sample. 

*S. pneumoniae*, *N. meningitidis* and *H. influenzae* were the most commonly reported isolates from bacterial meningitis cases in this study and similar other reports from Nepal [19,20,21,22,23]. Furthermore, meningitis caused by these bacteria are vaccine preventable. Therefore, the multiplex PCR targeting these three bacteria have been selected in this study. Our study showed that PCR could identify the etiology of few more bacterial meningitis cases compared to CSF culture. Considering culture as a gold standard test, we found PCR to be 100% sensitive and 98.87% specific. Our results are comparable with the similar other report [24]. Though considered as gold standard, the sensitivity of CSF culture is limited to 70–90% which presents a difficulty in evaluation of other potentially more sensitive tests [2].

PCR did not detect any additional cases of *N. meningitidis* or *S. pneumoniae*. However, similar to other researchers, we detected *H. influenzae* by PCR in four culture negative CSF samples [25]. This result also confirmed the detection of bacterial pathogens by acridine orange stain of the same CSF samples which were culture negative in our previous study (4). Fastidious bacteria are unlikely to survive outside of the host in the environment due to depletion of nutrients, fluctuations in temperature and relative humidity giving false negative culture results [26]. A low number of bacteria in the CSF samples might be missed by culture. On the other hand, bacterial DNA which is relatively stable and even in low number could be amplified and detected in the same sample by PCR [27]. Wang et al. using a real-time PCR reported that PCR was capable to identify the bacterial pathogen in 45% of cases while CSF culture detected the bacteria in only 9% of cases [28]. Thus, other PCR methods are likely more sensitive than the one we used. They required, however, special equipment not available in our laboratory and are much more expensive. Wu et al. concluded that PCR increases the yield of diagnosis for bacterial meningitis and should be incorporated into routine surveillance also in the low income countries [24]. However, compared to culture, PCR has the disadvantage of lacking the isolate on which conventional antibiotic susceptibility testing and additional strain characterization could be performed [29]. In this study, 75% of PCR-positive but culture-negative *H. influenzae* meningitis cases were under antibiotics administration prior to the collection of CSF samples. The detection of non-viable bacteria by PCR, the diminished sensitivity of culture in patients under antibiotic treatment and the inclusion of pediatric cases might explain the increased number of positivity in the detection of *H. influenzae*. PCR has been proved to be extremely useful in the early diagnosis of meningococcal meningitis even when the patient has received antibiotics prior to lumbar puncture. Kristiansen et al. concluded that meningococcal meningitis could be excluded on the basis of a negative PCR result [30]. However, our study showed no difference in detection of meningococci and pneumococci in CSF by both culture and PCR. 

CSF culture is relatively inexpensive and is still considered as the gold standard for the diagnosis of bacterial meningitis. Rapid methods of diagnosis like PCR can give life saving results. However, these methods also increase the cost of testing the CSF samples [31]. Moreover, the rapid testing results do not necessarily prompt the clinicians to alter empiric therapy in case of pediatric patients.

Laboratory diagnosis of bacterial meningitis in developing countries such as Nepal is based on two conventional methods—CSF culture and Gram’s stain. The four PCR positive *H. influenzae* cases would have been missed in our study by both the conventional methods i.e., CSF culture and Gram’s stain. However, the biochemical and cytological parameters indicated the characteristics of bacterial meningitis. Hence, available tests such as CSF cell count, glucose and protein concentration should also be considered while diagnosing the patient.

The major limitation of this study is that we focused only on three bacterial pathogens. Lack of testing for other bacteria causing meningitis such as Group B *Streptococcus*, *Listeria monocytogenes* and viruses might provide an explanation for the large number of negative samples obtained. However, even using advanced PCR technology, such as the multiplex PCR-based system provided by Film Array (BioFire, Biomérieux) which can detect simultaneously 14 meningitis pathogens, the etiology of meningitis was identified in only 10% of the suspected meningitis patients in Ethiopia [28]. 

The small number of PCR positive results in culture negative cases in our study might also be due to the case definition which is inadequate to differentiate between bacterial meningitis from aseptic meningitis. We included all the clinically suspected meningitis cases that met the clinical case definition. There is a chance that number of positive cases would have been increased if we used the case definition of probable meningitis cases. A probable meningitis case is a clinically suspected case with a turbid CSF (with or without positive Gram stain or ongoing epidemic and epidemiological link to a laboratory confirmed case [32]. Therefore, proper definition of cases also plays a major role in recruitment of cases.

## 5. Conclusions

PCR increases the diagnostic yield for bacterial meningitis. PCR may be considered as an adjunctive test for establishing the cause of infection in culture negative clinically suspected meningitis cases in developing countries.

## Figures and Tables

**Table 1:** Age and genderwise distribution of clinically suspected meningitis cases (*n* = 384).

Age Group	Female *n* (%)	Male *n* (%)	Total *n (%)*
Neonate < 1 month	72 (57.60)	53 (42.40)	125 (32.55)
Infant 1 month–1 year	29 (39.19)	45 (60.81)	74 (19.27)
Child 1–10 years	16 (40.00)	24 (60.00)	40 (10.42)
Adolescent 10–19 years	12 (36.36)	21 (63.64)	33 (8.59)
Adults 19–45 years	26 (32.50)	54 (67.50)	80 (20.83)
Adults above 45 years	11 (34.38)	21 (65.63)	32 (8.33)
Total	166 (43.23)	218 (56.77)	384

**Table 2:** Comparison of PCR and culture of cerebrospinal fluid (CSF) specimens for detection of *S. pneumoniae*, *N. meningitidis* and *H. influenzae* (*n* = 384).

Type of Test		Culture		Sensitivity	Specificity	Predictive Value	
		Positive	Negative			Positive	Negative
PCR	Positive	29	4	100%	98.87%	87.88%	100%
	Negative	0	351				

**Table 3:** Detection of bacteria in CSF of clinically suspected meningitis cases and antibiotics receipt prior to lumbar puncture (*n* = 384).

Bacteria	Culture No. (%)	PCR No. (%)	Antibiotics Receipt Prior to Lumbar Puncture	
			No. (% of Culture/PCR Positive)	Time Period Elapsed between Antibiotics Receipt and Culture/PCR
*Streptococcus pneumoniae*	12 (3.13)	12 (3.13)	1 (8.33)	2 days
*Neisseria meningitidis*	9 (2.34)	9 (2.34)	1 (11.11)	2 days
*Haemophilus influenzae*	8 (2.08)	12 (3.13)	3 (25%)	Case 1: 7 days Case 2: 6 days Case 3: 6 days
Total No. (%)	29 (7.55)	33 (8.59)	5 (15.15)	-

**Table 4:** Laboratory investigations of CSF samples from culture negative but PCR positive *H. influenzae* meningitis cases (*n* = 4).

Case No.	Gram’s Stain	Acridine Orange Stain	Glucose Concentration (mg/dL)	Protein Concentration (mg/dL)	Cell Count (WBCs/mm^3^)
1	Neg	Pos	15	170	500
2	Neg	Pos	25	120	800
3	Neg	Pos	30	135	200
4	Neg	Pos	32	130	200

## Data Availability

The data generated or analysed during this study are included in this published article.
